# APOBEC3 activity and DNA polymerase‐ε deficiency are associated with distinct *IDH1* R132 hotspot mutations

**DOI:** 10.1002/1878-0261.70334

**Published:** 2026-09-09

**Authors:** Kelly E. Butler, Bilal A. Lone, Ecem Unal, A. Rouf Banday

**Affiliations:** ^1^ Genitourinary Malignancies Branch, Center for Cancer Research National Cancer Institute, National Institutes of Health Bethesda MD USA

**Keywords:** APOBEC3s, cancer, DNA polymerase, genetics, IDH1, mutational signatures

## Abstract

*IDH1* R132 mutations are among the most frequent hotspot mutations in cancer, but their mutational origins have remained unclear. Here, we provide evidence that *IDH1* R132C, the predominant *IDH1* mutation in cholangiocarcinoma, acute myeloid leukemia, and melanoma, likely arises through APOBEC3‐mediated mutagenesis, specifically by APOBEC3A or APOBEC3B. *IDH1* R132C is a TpC>TpT substitution on the lagging‐strand DNA template within a hairpin‐forming sequence context, consistent with APOBEC3 susceptibility. *In vitro* assays showed that APOBEC3A and APOBEC3B can deaminate the relevant cytosine, while *APOBEC3A* and *APOBEC3B* expression patterns in tumor types with recurrent *IDH1* R132C were consistent with their potential involvement in generating this mutation. *IDH1* R132G, a TpC>TpG substitution at the same site, may similarly result from APOBEC3A or APOBEC3B activity. By contrast, *IDH1* R132H, the predominant *IDH1* mutation in lower grade glioma and glioblastoma, is a CpG>TpG substitution at a methylated cytosine on the leading‐strand DNA template, a pattern more consistent with DNA polymerase epsilon replication error. Concordantly, tumor types enriched for *IDH1* R132H showed relatively low *POLE* expression. Together, these *in vitro* and bioinformatic analyses provide insight into the distinct mutational mechanisms that likely underlie recurrent *IDH1* hotspot mutations in cancer.

## Graphical abstract

Isocitrate dehydrogenase 1 *(IDH1)* mutations are highly recurrent in multiple human cancer types, including cholangiocarcinoma and glioma. *IDH1* R132C is the most common *IDH1* mutation in cholangiocarcinoma and likely arises from APOBEC3A‐ or APOBEC3B‐mediated deamination. *IDH1* R132H is predominant in glioma and may arise from replication error by DNA polymerase epsilon.

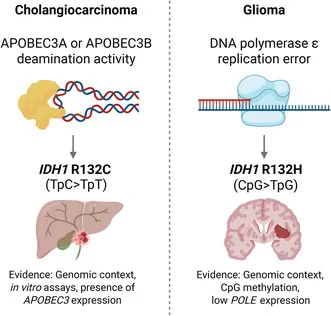

**Abbreviations**
APOBEC3s: apolipoprotein B mRNA‐editing enzyme, catalytic polypeptide threes
CHOL: cholangiocarcinoma
GBM: glioblastoma multiforme
IDH1: Isocitrate dehydrogenase 1
LAML: acute myeloid leukemia
LGG: lower grade glioma
MeDIP‐Seq: methylated DNA immunoprecipitation sequencing
RFD: replication fork directionality
SKCM: skin cutaneous melanoma
UDG: uracil DNA glycosylase

## 1 Introduction

Numerous distinct mutational processes contribute to carcinogenesis and leave characteristic mutational signatures, defined by the base substitution and surrounding sequence context [1]. One of the most prevalent endogenous mutational processes in cancer is mediated by apolipoprotein B mRNA‐editing enzyme catalytic polypeptide‐like threes (APOBEC3s), which are cytosine deaminases that facilitate innate antiviral defense [2]. However, APOBEC3s are frequently overexpressed in cancer and can deaminate host DNA, thereby generating oncogenic mutations [2]. APOBEC3s preferentially deaminate cytosines in single‐stranded DNA, such as within hairpin loops and lagging strand DNA templates during replication [3, 4, 5, 6, 7]. APOBEC3‐mediated cytosine deamination generates uracil, and subsequent erroneous repair or replication of the uracil‐containing template generates C>T or C>G mutations [2]. These mutations underlie the COSMIC single‐base substitution signatures SBS2 and SBS13, respectively [8]. While mutations in TCW (W = A or T) motifs are most common, APOBEC3s can also deaminate cytosines in TCG and TCC motifs, including for known driver mutations [9, 10, 11, 12]. Among the seven APOBEC3s, APOBEC3A and APOBEC3B are most strongly associated with SBS2 and SBS13 [2, 8]. APOBEC3G is also mutagenic, but it generates a different mutational signature predominantly characterized by C>T mutations in CC sites [13].

Another common mutational process that generates C>T substitutions is spontaneous deamination of 5‐methylcytosine, often within CpG sites [8, 14]. This process is tightly linked with aging and classically represented by the ‘clock‐like’ signature SBS1 [8, 14]. However, recent studies have identified DNA polymerase epsilon error as an additional potential source of C>T mutations at methylated CpG sites [15, 16]. DNA polymerase epsilon carries out leading‐strand DNA synthesis, and erroneous incorporation of adenine opposite 5‐methylcytosine can generate C>T transitions independent of other polymerases or repair pathways [15, 16]. The proofreading fidelity of DNA polymerase epsilon decreases with template‐strand methylation, making replication errors particularly frequent at CpG sites [15, 16]. DNA polymerase epsilon exonuclease domain mutations are canonically associated with SBS10 and SBS14 [1, 8]. Wild‐type DNA polymerase epsilon may also generate CpG>TpG mutations and contribute to additional mutational signatures, although its error rate is substantially lower than that of mutant DNA polymerase epsilon [15, 16]. Studies in yeast further suggest that reduced expression of wild‐type DNA polymerase epsilon may create a mutator phenotype with increased probability of replication error [17]. In addition, DNA polymerase delta, which replicates the lagging strand, has been shown to play a role in repairing replication errors made by DNA polymerase epsilon [18].

The mutational processes underlying recurrent alterations in isocitrate dehydrogenase 1 (*IDH1*) have not yet been characterized. *IDH1* mutations are among the most recurrent mutations in cancer and occur in multiple tumor types including glioma, cholangiocarcinoma, and acute myeloid leukemia [19]. IDH1 is a metabolic enzyme that normally generates α‐ketoglutarate [20]. In cancer, gain‐of‐function *IDH1* mutations lead to production of the oncometabolite 2‐hydroxyglutarate (2‐HG) [19, 20, 21]. Since 2‐HG cannot be used in major metabolic pathways, it accumulates in *IDH1*‐mutated tumor cells and causes metabolic, epigenetic, and signaling alterations that promote tumorigenesis and allow immune evasion [19, 22, 23, 24]. For example, 2‐HG‐induced global hypermethylation may block differentiation of *IDH1*‐mutated tumor cells [22, 24]. *IDH1* mutations occur early in tumorigenesis and are therapeutically targeted driver mutations [19, 25]. Small‐molecule inhibitors targeting mutated IDH1 protein are currently used in clinical practice and include ivosidenib, olutasidenib, and vorasidenib (targets mutated IDH1/2) [25, 26, 27, 28, 29, 30, 31, 32].

Given the biological and clinical significance of *IDH1* mutations, it is important to understand their molecular etiology. Here, we evaluated whether recurrent *IDH1* mutations are aligned with known mutational signatures. Based on *in vitro* and computational analyses, we propose that *IDH1* R132C and R132G likely arise from APOBEC3A or APOBEC3B activity, while *IDH1* R132H may result from DNA polymerase epsilon error.

## 2 Materials and methods

### 2.1 Analysis of TCGA data

Mutation, gene expression, and DNA methylation data for tumors in the TCGA Pan‐Cancer Atlas were accessed through cBioPortal [33, 34, 35]. Gene expression data for normal bile duct tissue adjacent to cholangiocarcinoma tumors were accessed through UCSC Xena; comparable adjacent normal data were not available for the other tumor types examined in this study [36]. All gene expression data were derived from Illumina HiSeq RNASeqV2. DNA methylation data were derived from merged HM27 and HM450 Illumina microarrays. Clinical data, specifically age at diagnosis, were accessed through cBioPortal. All data were accessed in 2025. Data visualization and statistical analyses were completed using graphpad prism.

### 2.2 Analysis of replication fork directionality using OK‐seq data

Replication fork directionality (RFD) at the *IDH1* locus was analyzed using OK‐seq data from HeLa S3 cells obtained from GEO (GSE193547). Genome‐wide continuous RFD signals were processed with the OKseqHMM pipeline and aligned to the human reference genome hg19, using the bigWig file GSM5814025_Hela_S3_all_cutadapt_sort_delDupl_merge_hmm_RFD_cutoff10_bs1kb_sort.bw. RFD values across the *IDH1* locus (chr2:209098953–209 121 795) were extracted from the bigWig file using a custom python workflow. Data extraction was performed with pybigwig (v0.3.23), and downstream processing was carried out in pandas (v2.0.3). Visualization was generated in matplotlib (v3.7.2), using the midpoint of each genomic interval to plot a continuous RFD profile. For genome‐browser visualization, the RFD bigWig track was loaded into Integrative Genomics Viewer (IGV; v2.17.4).

### 2.3 Cell culture

The human embryonic kidney 293FT cell line (R70007) was purchased from Thermo Fisher Scientific (Waltham, MA, USA). The human bladder cancer cell line HT1376 (CRL‐1472) was obtained from the American Type Culture Collection (ATCC; Manassas, VA, USA). Both cell lines were authenticated by the supplier and used within 2 years of purchase. These cell lines are listed in Cellosaurus as HEK293‐FT (RRID: CVCL_6911) and HT1376 (RRID: CVCL_1292). All cell lines were routinely tested for mycoplasma contamination using the MycoAlert® Mycoplasma Detection Kit (LT07‐318; Lonza, Basel, Switzerland), and experiments were performed using mycoplasma‐free cells. Cells were maintained at 37 °C in a humidified incubator with 5% CO_2_. HT1376 cells were cultured in Eagle's Minimum Essential Medium (EMEM; ATCC 30‐2003) supplemented with 10% fetal bovine serum (FBS). 293FT cells were cultured in high‐glucose Dulbecco's Modified Eagle Medium (DMEM) supplemented with 10% FBS.

### 2.4 *In vitro* APOBEC3A and APOBEC3B DNA cytosine deamination assays

Deamination assays were performed using APOBEC3A and APOBEC3B as previously described, with minor modifications [37, 38]. First, plasmids encoding Myc‐tagged human APOBEC3A (#RC220995; OriGene) or APOBEC3B (#RC222712; OriGene Technologies, Rockville, MD, USA) were transfected into 293FT cells using Lipofectamine LTX. APOBEC3A was also transfected into HT1376 cells. Cells were grown in 10‐cm plates. Following two days of incubation, APOBEC3A or APOBEC3B protein was isolated using the c‐Myc tagged protein MILD PURIFICATION KIT Ver.2 (#3305; MBL Life Science, Nagoya, Japan). For the *in vitro* deamination assays, partially purified APOBEC3A protein (0.25 μg) or APOBEC3B protein (40 μg) was incubated for 1 h (APOBEC3A from 293FT cells), 30 min (APOBEC3A from HT1376 cells), or 3 h (APOBEC3B from 293FT cells) at 37 °C in 10 μL reaction mixtures consisting of the following components: 20 pmol of custom DNA oligonucleotide probe (synthesized by ‐ Integrated DNA Technologies, Coralville, IA, USA), 10 mm Tris–HCl (pH 8.0), 50 mm NaCl, and 1 mm DTT. For reactions with APOBEC3B, ZnCl_2_ was added at a final concentration of 5 μm. A higher amount of partially purified APOBEC3B protein was used because APOBEC3B was recovered at a substantially lower fraction of total protein than APOBEC3A using this affinity‐purification approach, as previously described [37]. Next, 5 μL of uracil DNA glycosylase (UDG) master mix, containing 0.75 units of UDG, was added to each reaction and incubated at 37 °C for 40 min. Subsequently, 5 μL of 0.6 N NaOH was added to each tube and incubated at 37 °C for an additional 20 min. An equal volume (20 μL) of 2× TBE‐Urea Sample Buffer (Invitrogen, Carlsbad, CA, USA) was then added, and the samples were heated at 95 °C for 3 min, then cooled on ice for 5 min. DNA cleavage was analyzed using a 15% TBE‐Urea polyacrylamide gel (Invitrogen), resolved at 100 V for 2 h at room temperature in 1× TBE buffer. The gel images were captured using the Li‐COR Odyssey imaging system with Alexa 488 fluorescence settings.

The custom oligonucleotide probes were single‐stranded DNA sequences centered at the cytosines mutated in *IDH1* R132C and *IDH1* R132H. Two sets of probes for each mutation were used: ‘a probes’ had nearby cytosines replaced with guanosines to avoid non‐specific deamination, and ‘b probes’ were wild‐type sequences.

Positive control: 5′‐ 6FAM‐ATGATTATTATT **C** CCAATTATTTGT‐3′.

R132C‐a (minus strand): 5′‐ 6FAM‐ATCAT ***G*** ATAGGT **C** GT ***G*** ATGCTTATG‐3′.

R132C‐b (minus strand): 5′‐ 6FAM‐ATCATCATAGGT **C** GTCATGCTTATG‐3′.

R132H‐a (plus strand): 5′‐ 6FAM‐CCATAAG ***G*** ATGA **C** GA ***GG*** TATGATGA‐3′.

R132H‐b (plus strand): 5′‐ 6FAM‐CCATAAGCATGA **C** GACCTATGATGA‐3′.

### 2.5 Analysis of CpG methylation at the *IDH1* R132H locus

Methylated DNA immunoprecipitation (MeDIP) data for brain tissue were visualized in UCSC Genome Browser using the UCSF Brain DNA Methylation track for hg19 [39, 40]. Visualization focused on the CpG locus corresponding to the cytosine altered in *IDH1* R132H, on the plus strand of DNA. The genomic region was also evaluated for the presence or absence of CpG islands using the UCSC Genome browser CpG Island Track.

## 3 Results

### 3.1 Recurrent *IDH1* R132 hotspot mutations involve adjacent cytosines on opposite DNA strands

**Fig. 1:**
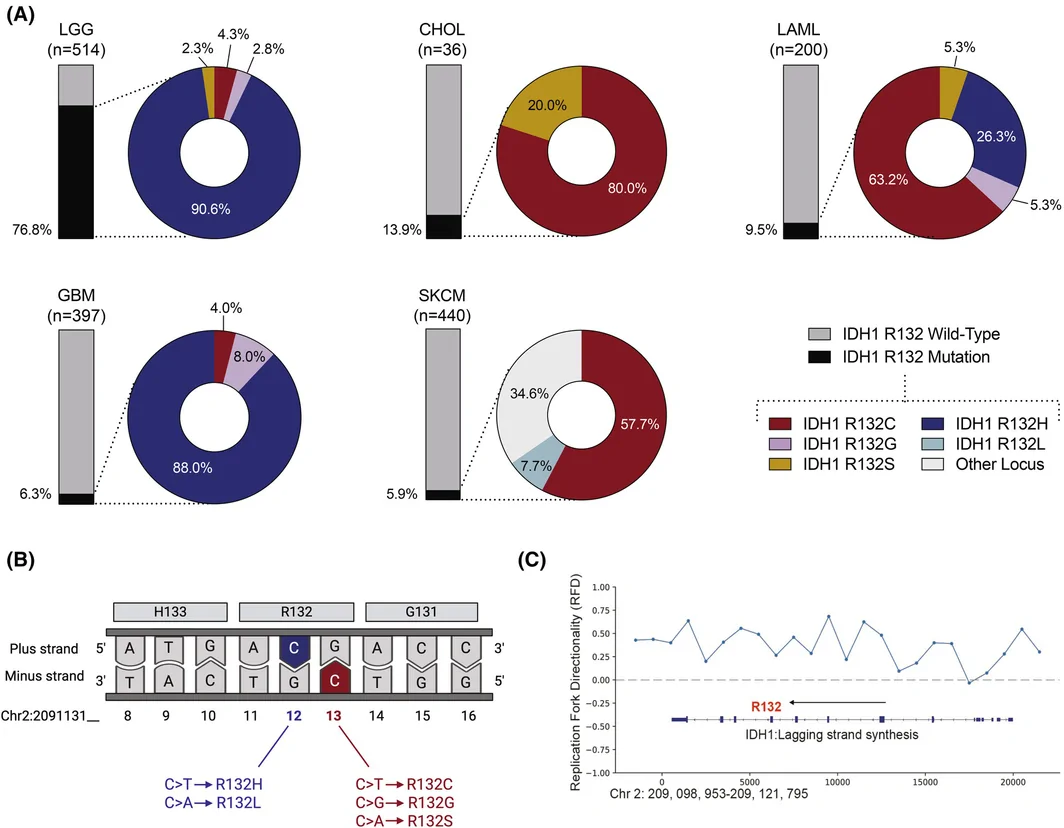
Recurrent *IDH1* R132 mutations map to adjacent cytosines on opposite DNA strands. (A) *IDH1* mutation frequency in cancer types with at least 5% *IDH1* mutation rate in the TCGA Pan‐Cancer Atlas dataset: lower grade glioma (LGG), cholangiocarcinoma (CHOL), acute myeloid leukemia (LAML), glioblastoma multiforme (GBM), and skin cutaneous melanoma (SKCM). (B) Sequence context and nucleotide substitutions for recurrent *IDH1* R132 mutations. (C) DNA replication fork directionality (RFD) for *IDH1* in the context of the minus strand. Positive RFD values indicate lagging strand synthesis.

*IDH1* mutations predominantly occur at amino acid position R132 and are recurrent in multiple cancer types including lower grade glioma (76.8%), cholangiocarcinoma (13.9%), acute myeloid leukemia (9.5%), glioblastoma (6.3%), and skin cutaneous melanoma (5.9%) (Fig. 1A). *IDH1* R132H is most recurrent in lower grade glioma and glioblastoma, while *IDH1* R132C is the most common *IDH1* mutation in cholangiocarcinoma, acute myeloid leukemia, and melanoma. *IDH1* R132G, R132L, and R132S occur at lower frequencies. All five *IDH1* R132 mutations arise from mutated cytosines at Chr2 : 209113112 or Chr2 : 209113113 (hg19), which are adjacent nucleotide positions on opposite DNA strands (Fig. 1B).

### 3.2 *IDH1* R132C and R132G likely arise from APOBEC3A or APOBEC3B activity

**Fig. 2:**
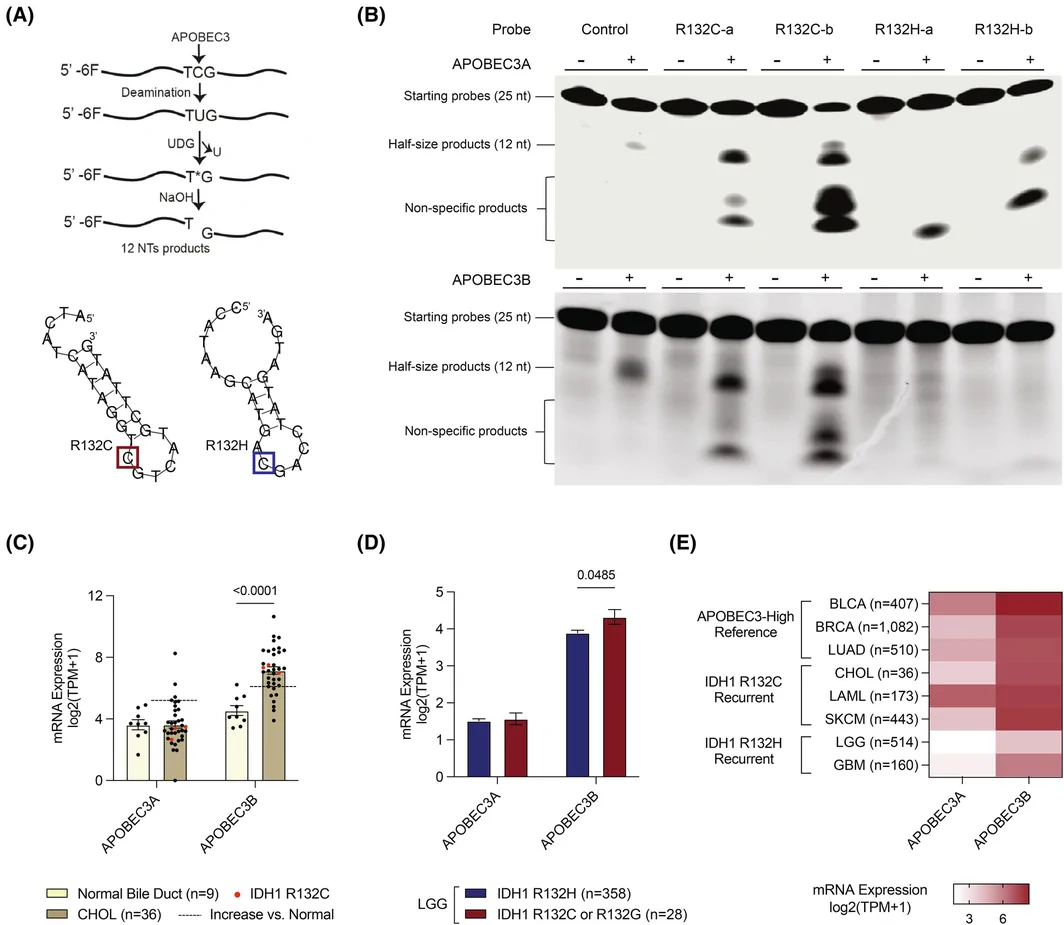
APOBEC3A or APOBEC3B activity may generate *IDH1* R132C and *IDH1* R132G. (A) Schematic of *in vitro* deamination assay (upper), and visualization of DNA secondary structures for deamination assay probes (lower). Visualized probes are ‘b‐probes’, which are wild‐type sequences centered at the recurrently mutated cytosines for *IDH1* R132C and *IDH1* R132H. Starting probes are 25 nucleotides (nt). Half‐size (12 nt) products indicate APOBEC3‐mediated deamination at the central cytosine of interest. (B) *In vitro* deamination assays for recurrently mutated *IDH1* loci using APOBEC3A (upper) or APOBEC3B (lower) from 293FT cells. Results are representative of multiple independent experiments (*n* = 3). (C) *APOBEC3A* and *APOBEC3B* mRNA expression in normal bile duct tissue and cholangiocarcinoma (CHOL). Tumors harboring *IDH1* R132C are indicated in red. Data are shown as mean ± SEM. Thresholds for significant increases in *APOBEC3A* and *APOBEC3B* expression are shown as horizontal lines, calculated as the one‐sided 95% confidence interval relative to normal bile duct tissue. Statistical analyses of *APOBEC3A* and *APOBEC3B* expression in CHOL versus normal bile duct tissue were conducted using Welch's *t*‐tests; *P* < 0.0001 for *APOBEC3B*. (D) *APOBEC3A* and *APOBEC3B* expression in lower grade glioma (LGG) tumors with *IDH1* R132C or R132G versus *IDH1* R132H. Data are shown as mean ± SEM. Statistical analyses were conducted using Welch's *t*‐tests; *P* = 0.0485 for *APOBEC3B*. (E) Heatmap showing mean *APOBEC3A* and *APOBEC3B* expression in cancer types with recurrent *IDH1* mutation, grouped by predominant *IDH1* mutation (R132C or R132H). Data are also shown for three cancer types known to have markedly high APOBEC3‐associated mutational burdens: bladder cancer (BLCA), breast cancer (BRCA), and lung adenocarcinoma (LUAD).

*IDH1* R132C and R132G are C>T and C>G mutations, respectively, within a TpCpN motif, a sequence context compatible with the APOBEC3A‐ and APOBEC3B‐associated mutational signatures SBS2 and SBS13 (Fig. 1B). Analysis of replication fork directionality using OK‐Seq data showed that the cytosine altered in *IDH1* R132C and R132G occurs on the lagging strand template (Fig. 1C). The *IDH1* R132C/R132G site also occurs within a region capable of forming a hairpin loop (Fig. 2A). Together, these structural features make the TpCpN mutation context a permissive substrate for APOBEC3A and APOBEC3B activity. To test this functionally, we performed *in vitro* deamination assays using APOBEC3A and APOBEC3B protein. Our assays demonstrated that both APOBEC3A and APOBEC3B can deaminate the cytosines within the *IDH1* R132C/R132G substitution context (Fig. 2B and Fig. S1). In contrast, the cytosine altered in *IDH1* R132H was not subject to *in vitro* deamination by either APOBEC3A or APOBEC3B, likely because it does not occur within a TpCpN context susceptible to APOBEC3s (Figs 1B, 2B, and Fig. S1).

We next examined expression patterns of *APOBEC3* family members in tumor tissues. *APOBEC3B* was significantly upregulated in cholangiocarcinoma—the studied tumor type in which *IDH1* R132C is most recurrent—compared to normal bile duct tissue (Fig. 2C). In cholangiocarcinoma, *IDH1* R132C occurred exclusively in tumors with elevated *APOBEC3B* expression relative to normal bile duct tissue (Fig. 2C). Although *APOBEC3A* expression was not strongly elevated, it was clearly detectable in both normal bile duct tissue and cholangiocarcinoma (mean log_2_ (TPM + 1) ~ 3.6; Fig. 2C). In lower grade glioma, tumors harboring *IDH1* R132C exhibited significantly higher A*POBEC3B* expression than tumors with *IDH1* R132H, the more common *IDH1* mutation in this tumor type (Fig. 2D).

In a cross‐cancer comparison, *APOBEC3A* and *APOBEC3B* expression were higher in tumor types with more recurrent *IDH1* R132C mutations—specifically cholangiocarcinoma, acute myeloid leukemia, and melanoma—than in tumor types with more recurrent *IDH1* R132H mutations, namely lower grade glioma and glioblastoma (Fig. 2E). *APOBEC3A* and *APOBEC3B* expression in tumor types with predominant *IDH1* R132C were also comparable to that observed in cancers known to have markedly high SBS2 and SBS13 mutational burdens, including breast cancer (Fig. 2E). Within individual cancer types, tumors with *IDH1* R132C or R132G did not exhibit higher *APOBEC3A* or *APOBEC3B* expression than tumors with wild‐type *IDH1* (Fig. S2A). This may partly reflect *IDH1* mutation–associated epigenetic remodeling, as increased methylation at cg22954818, located within a regulatory region upstream of the APOBEC3 gene cluster near the *APOBEC3A* promoter, was observed in *IDH1*‐mutated tumors versus *IDH1* wild‐type tumors (Fig. S2B).

Since the cytosine altered in *IDH1* R132C and R132G occurs within a CpG site, we also evaluated age‐related spontaneous deamination as a potential etiology. However, this is highly unlikely because IDH1 R132C and R132G were not associated with older age in any cancer type (Fig. S2C). Together with the sequence‐context, strand‐orientation, *in vitro* deamination, and gene expression analyses, the lack of an age association for *IDH1* R132C and R132G further supports an APOBEC3‐mediated mutational etiology.

### 3.3 *IDH1* R132H may arise from DNA polymerase epsilon error

**Fig. 3:**
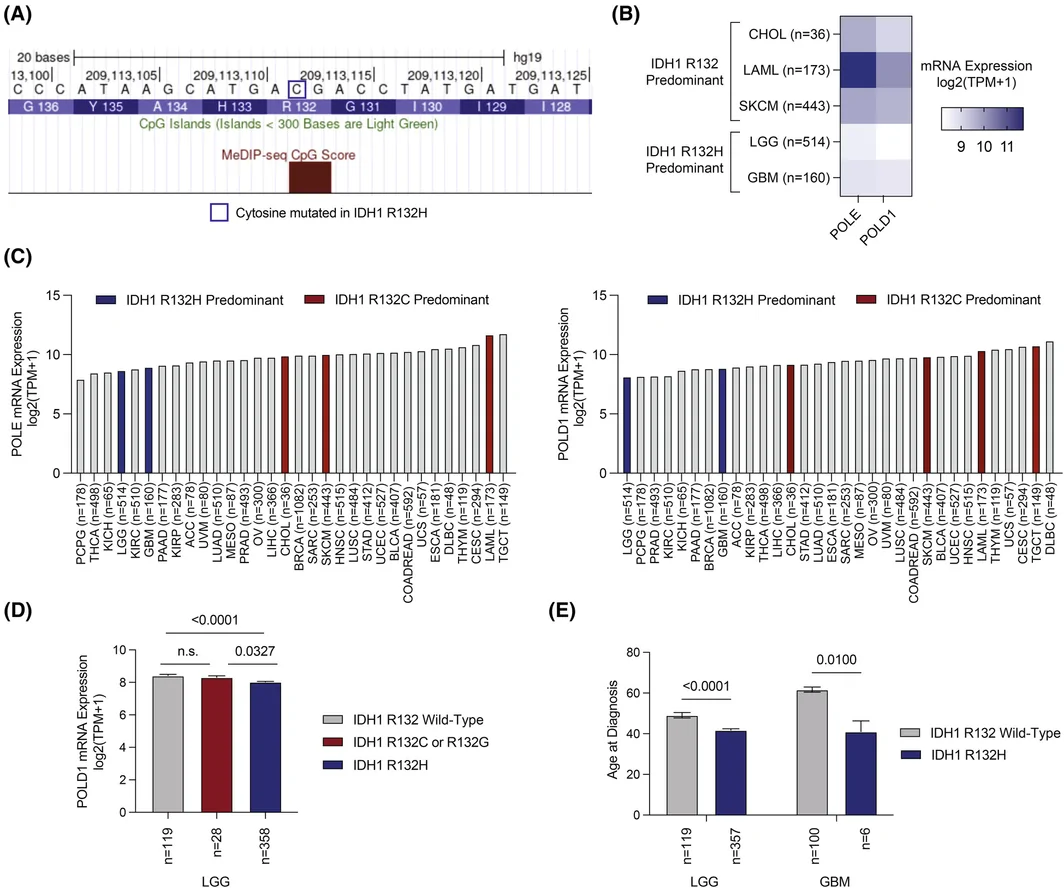
DNA polymerase epsilon error is a possible etiology for *IDH1* R132H. (A) Methylated DNA immunoprecipitation (Me‐DIP) CpG score at the *IDH1* R132H mutation locus, visualized on the plus strand of DNA using a dataset derived from brain tissue. A higher score indicates methylation. CpG islands were not present at the locus. (B) Heatmap showing mean *POLE* and *POLD1* mRNA expression in cancer types with recurrent *IDH1* mutation: cholangiocarcinoma (CHOL), acute myeloid leukemia (LAML), skin cutaneous melanoma (SKCM), lower grade glioma (LGG), and glioblastoma multiforme (GBM). Cancer types are grouped by predominant *IDH1* mutation (R132C or R132H). (C) *POLE* (left) and *POLD1* (right) mRNA expression in all tumor types in the TCGA Pan‐Cancer Atlas dataset. Cancer type abbreviations use standard TCGA nomenclature. Data are shown as means. (D) *POLD1* mRNA expression in LGG tumors by *IDH1* mutation status (*IDH1* wild‐type versus *IDH1* R132C or R132G versus *IDH1* R132H). Data are shown as mean ± SEM. Statistical analyses were conducted using post hoc Dunn's tests adjusted for multiple comparisons; *P* < 0.0001 for *IDH1* R132 wild‐type versus *IDH1* R132H, *P*‐value non‐significant (ns) for *IDH1* R132 wild‐type versus *IDH1* R132C or R132G, *P* = 0.0327 for *IDH1* R132C or R132G versus *IDH1* R132H. (E) Age at diagnosis for patients with *IDH1* wild‐type versus *IDH1* R132H‐mutated LGG or GBM. Data are shown as mean ± SEM. Statistical analyses were conducted using Welch's *t*‐tests; *P* < 0.0001 for LGG, *P* = 0.0100 for GBM. Age data were not available for LAML.

*IDH1* R132H is a CpG>TpG mutation within leading‐strand DNA, which raised the possibility that it arises from DNA polymerase epsilon error (Fig. 1B,C). Concordant with this suspected etiology, analysis of methylated DNA immunoprecipitation (Me‐DIP) data revealed that the relevant cytosine is methylated in brain tissue—an epigenetic modification consistent with its location in a coding exon rather than a regulatory CpG island (Fig. 3A). Methylation data from brain tissue were analyzed because *IDH1* R132H is most common in lower grade glioma and glioblastoma.

Compared to tumor types with predominant *IDH1* R132C, lower grade glioma and glioblastoma had relatively low expression of *POLE* and *POLD1*, which encode the catalytic subunits for DNA polymerase epsilon and delta, respectively (Fig. 3B). Glioma and glioblastoma also had among the lowest *POLE* and *POLD1* expression levels across all tumor types, with lower grade glioma having the least *POLD1* expression of any cancer (Fig. 3C). Among lower grade gliomas, *IDH1* R132H‐mutated tumors had significantly reduced *POLD1* expression compared to tumors with wild‐type *IDH1*; *POLD1* expression was also reduced in *IDH1* R132H‐mutated gliomas compared to *IDH1* R132C‐ or R132G‐mutated gliomas (Fig. 3D). Taken together, these data suggest that DNA polymerase epsilon error in the setting of low POLE expression could lead to the initial genomic insult underlying *IDH1* R132H, while low *POLD1* expression may contribute to the mutation remaining unrepaired.

We also considered spontaneous deamination of 5‐methylcytosine as an alternative cause of *IDH1* R132H, as this mutational process often causes CpG>TpG mutations of methylated cytosines. However, this mutational etiology is unlikely because it is age‐associated unlike *IDH1* R132H mutations. In both lower grade glioma and glioblastoma, *IDH1* R132H was associated with younger age at diagnosis (Fig. 3E).

## 4 Discussion

**Fig. 4:**
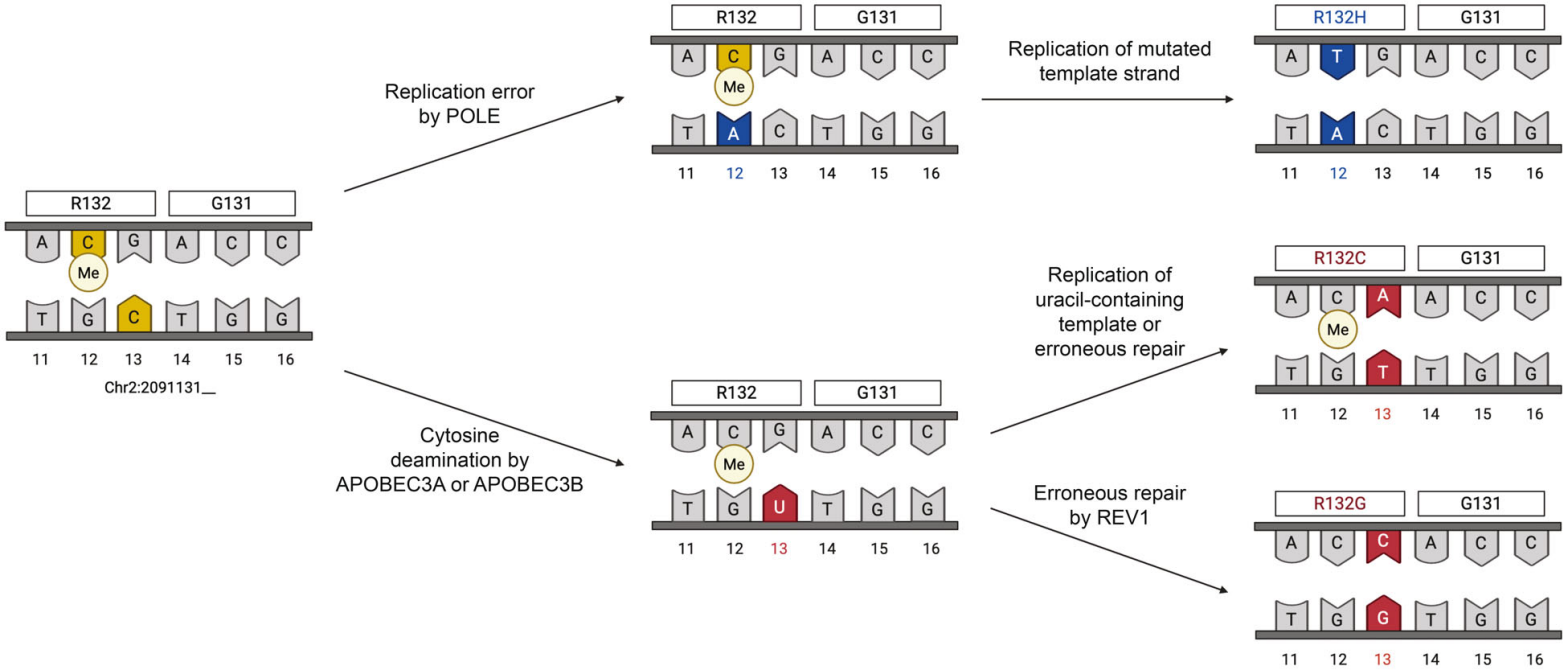
Summary of likely etiologies for *IDH1* R132C, R132G, and R132H mutations. Different *IDH1* R132 mutations likely have distinct etiologies. DNA polymerase epsilon error during replication of leading‐strand DNA may generate *IDH1* R132H (CpG>TpG) mutations. In contrast, *IDH1* R132C (TpC>TpT) and R132G (TpC>TpG) may arise from APOBEC3A‐ and/or APOBEC3B‐mediated deamination of lagging‐strand DNA, generating an intermediate uracil‐containing template; distinct replication or repair pathways can then generate C>T and C>G mutations.

Overall, our results implicate distinct mutational processes as likely sources of different oncogenic *IDH1* R132 alleles (Fig. 4). We first propose that APOBEC3A and/or APOBEC3B activity can generate *IDH1* R132C and R132G based on (a) concordance with SBS2 and SBS13 and presence of genomic features that may increase susceptibility to APOBEC3s, (b) presence of *APOBEC3A* and *APOBEC3B* expression in tumor types with predominant IDH1 R132C, and (c) direct *in vitro* evidence that APOBEC3A and APOBEC3B can deaminate the relevant cytosine. The higher frequency of *IDH1* R132C relative to *IDH1* R132G may reflect the predominance of C>T over C>G outcomes following APOBEC3‐mediated deamination and functional differences in the resulting amino acid substitutions; for example, IDH1 R132G produces a larger quantity of 2‐HG that may be cytotoxic to tumor cells [2, 8, 41, 42]. C>A mutations can even more rarely arise from APOBEC3 activity, so it is possible that *IDH1* R132S also arises from APOBEC3‐mediated deamination of the same cytosine [1, 8].

*APOBEC3A* and *APOBEC3B* expression in cholangiocarcinoma, acute myeloid leukemia, and melanoma may help explain why R132C is the most common *IDH1* mutation in these tumor types (1). *IDH1* R132C and R132G are also common in chondrosarcoma, a cancer type that was not represented in the TCGA dataset [19]. In cholangiocarcinoma, established risk factors—such as hepatitis infection and inflammatory conditions (e.g., primary sclerosing cholangitis)—could plausibly promote APOBEC3 induction, as *APOBEC3s* are interferon‐stimulated genes upregulated by JAK‐STAT and NF‐κB signaling [2, 43]. Interestingly, we did not observe *IDH1* R132C or R132G mutations in other tumor types with high APOBEC3 activity (e.g., bladder and breast cancers), suggesting tissue‐specific differences in the selective advantage conferred by IDH1 R132 variants; similarly, other APOBEC3‐associated hotspots, such as FGFR3 S249C, are highly tumor‐type specific [11]. Notably, the APOBEC3A‐ and APOBEC3B‐associated signatures SBS2 and SBS13 have been documented in cholangiocarcinoma but are minimally present in glioma and glioblastoma, which much more frequently harbor *IDH1* R132H rather than R132C [1, 44]. These patterns are consistent with our hypothesis that APOBEC3A and/or APOBEC3B may cause *IDH1* R132C and IDH1 R132G mutations in cholangiocarcinoma, while a different mutational process induces *IDH1* R132H in glioma and glioblastoma.

Finally, our data raise the possibility that *IDH1* R132H may result from DNA polymerase epsilon error, which is plausible since the relevant cytosine occurs on the leading‐strand template and is methylated. Comparatively low *POLE* expression in lower grade glioma and glioblastoma may help explain the predominance of *IDH1* R132H in these tumor types. It remains unclear whether low *POLE* expression emerges during tumorigenesis or reflects a baseline feature of healthy glial cells, as gene‐expression data from adjacent normal tissue were not available for lower grade glioma and glioblastoma. However, it is plausible that the minimal baseline turnover of healthy glial cells is accompanied by lower DNA polymerase epsilon expression, thereby creating a vulnerability to replication error during infrequent cell division events (e.g., reactive gliosis, NG2+ cell renewal) [45, 46, 47, 48]. This paradigm is consistent with *IDH1* R132 mutations arising early in tumorigenesis.

More broadly, multiple distinct mutational processes acting on the same codon to generate different amino acid substitutions likely reflect strong oncogenic selection pressure, underscoring the important role of *IDH1* R132 mutations in driving tumorigenesis across multiple cancer types.

## 5 Conclusions

The molecular etiology of *IDH1* R132 mutations has remained unknown. Based on analysis of genomic features, gene expression data, and *in vitro* deamination assays, we propose that APOBEC3A and/or APOBEC3B activity may give rise to *IDH1* R132C and R132G mutations. In contrast, *IDH1* R132H may arise from replication error by DNA polymerase epsilon. These findings provide insight into the molecular oncogenesis of multiple cancer types, including cholangiocarcinoma and glioma.

## Conflict of interest

The authors declare no conflict of interest.

## Author contributions

KEB and ARB conceptualized the study design and wrote the manuscript. KEB and EU completed bioinformatic analyses. BAL conducted the *in vitro* deamination assays. All authors contributed to writing the materials and methods section and reviewed the final manuscript.

## Supporting information


**Fig. S1.** *In vitro* deamination assay for recurrently mutated *IDH1* loci using APOBEC3A expressed in HT1376 cells. Single‐stranded DNA probes were 25 nucleotides (nt) in length, centered around the cytosines altered in *IDH1* R132C/R132G or *IDH1* R132H. Reactions were performed in the absence or presence of APOBEC3A, followed by uracil DNA glycosylase (UDG) exposure and alkaline cleavage. The positive control probe showed the expected half‐size (12 nt) cleavage product. APOBEC3A deaminated probes containing the *IDH1* R132C/R132G cytosine context, generating the expected half‐size cleavage products. APOBEC3A did not deaminate the *IDH1* R132H probe. Non‐specific cleavage products are indicated. Results are representative of multiple independent experiments (*n* = 3).

**Fig. S2.** *APOBEC3* expression, *APOBEC3* methylation, and patient age at diagnosis by *IDH1* mutation status. (A) *APOBEC3A* (left) and *APOBEC3B* (right) expression in IDH1 wild‐type versus IDH1 R132C‐ or R132G‐mutated tumors in lower grade glioma (LGG), cholangiocarcinoma (CHOL), acute myeloid leukemia (LAML), and skin cutaneous melanoma (SKCM). Glioblastoma multiforme (GBM) is not shown due to limited data availability for IDH1 R132C‐ and R132G‐mutated tumors. Data are shown as mean ± SEM. Statistical analyses were conducted using Welch's *t* tests. (B) Methylation of cg22954818, which is upstream of the *APOBEC3* gene cluster in a regulatory region for *APOBEC3A*, in IDH1 wild‐type versus *IDH1* R132‐mutated tumors for LGG, CHOL, LAML, GBM, and SKCM. Tumors with any IDH1 R132 mutation were included in the analysis because 2‐HG‐induced hypermethylation is not known to be allele‐specific. Data are based on merged HM27 and HM450 Illumina microarrays. Data are shown as mean ± SEM. Statistical analyses were conducted using Welch's *t*‐tests; *P* < 0.0001 for LGG, *P* = 0.0016 for LAML, *P* = 0.0134 for GBM. (C) Age at diagnosis for patients with IDH1 wild‐type versus IDH1 R132C‐ or IDH1 R132G‐mutated LGG, CHOL, or SKCM. Age data were not available for LAML, or for GBM tumors with IDH1 R132C or R132G mutations. Data are shown as mean ± SEM. Statistical analyses were conducted using Welch's *t* tests; *P* = 0.0001 for LGG.

## Data Availability

Computational analyses used the following publicly available datasets: TCGA Pan Cancer Atlas (accessed through cBioPortal and UCSC Xena), OKseqHMM: A Genome‐Wide Replication Fork Directionality Analysis Toolkit (accessed through GEO, accession number GSE193547), UCSF Brain DNA Methylation Track (accessed through UCSF Genome Browser), and the CpG Islands Track (accessed through UCSF Genome Browser). OKseq dataset: https://www.ncbi.nlm.nih.gov/geo/query/acc.cgi?acc=GSE193547, Brain DNA Methylation Track: https://genome.ucsc.edu/cgi-bin/hgTables?db=hg19&hgta_group=regulation&hgta_track=ucsfBrainMethyl&hgta_table=ucsfChipSeqH3K4me3BrainCoverage&hgta_doSchema=describe+table+schema, CpG Island Track: https://genome.ucsc.edu/cgi-bin/hgTrackUi?hgsid=4143698755_AgCTudTznfGIXhTeciBR0adiSNSQ&db=hg19&c=chr2&g=cpgIslandSuper.
