# Supplementary material for: APOBEC3 activity and DNA polymerase‐ε deficiency are associated with distinct IDH1 R132 hotspot mutations
Source: Mol Oncol. 2026 Sep 9:10.1002/1878-0261.70334. Online ahead of print. doi: 10.1002/1878-0261.70334 (PMC13558905; doi:10.1002/1878-0261.70334)
Supplement: Supplementary file 1 [file MOL2-9999-0-s001.pdf]

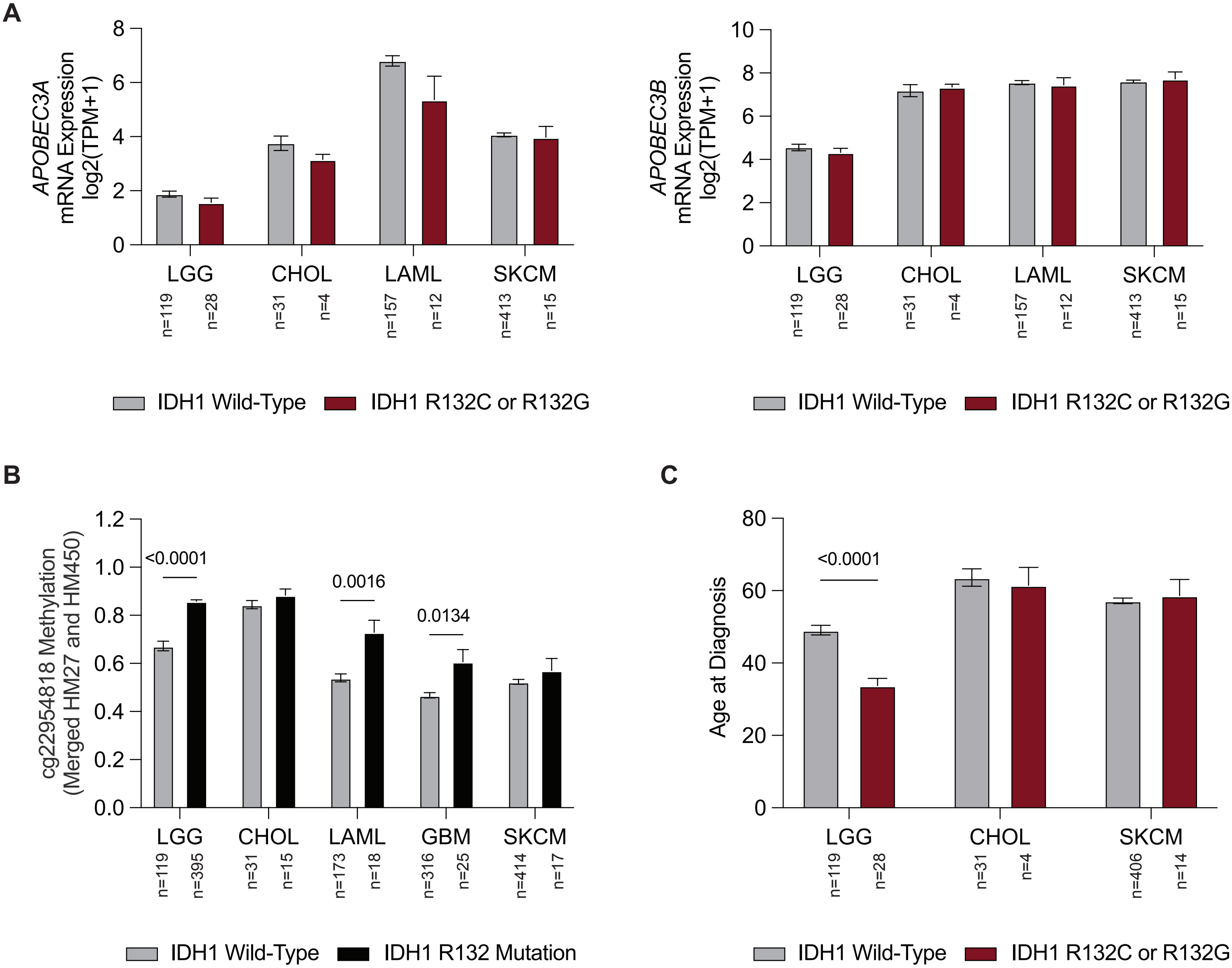
A
APOBEC3A mRNA Expression log2(TPM+1)
0
2
4
6
8
LGG
CHOL
LAML
SKCM
n=119
n=28
n=31
n=4
n=157
n=12
n=413
n=15
IDH1 Wild-Type
IDH1 R132C or R132G
APOBEC3B mRNA Expression log2(TPM+1)
0
2
4
6
8
10
LGG
CHOL
LAML
SKCM
n=119
n=28
n=31
n=4
n=157
n=12
n=413
n=15
IDH1 Wild-Type
IDH1 R132C or R132G
B
cg22954818 Methylation (Merged HM27 and HM450)
0.0
0.2
0.4
0.6
0.8
1.0
1.2
<0.0001
0.0016
0.0134
LGG
CHOL
LAML
GBM
SKCM
n=119
n=395
n=31
n=15
n=173
n=18
n=316
n=25
n=414
n=17
IDH1 Wild-Type
IDH1 R132 Mutation
C
Age at Diagnosis
0
20
40
60
80
<0.0001
LGG
CHOL
SKCM
n=119
n=28
n=31
n=4
n=406
n=14
IDH1 Wild-Type
IDH1 R132C or R132G
