# Supplementary material for: APOBEC3 activity and DNA polymerase‐ε deficiency are associated with distinct IDH1 R132 hotspot mutations
Source: Mol Oncol. 2026 Sep 9:10.1002/1878-0261.70334. Online ahead of print. doi: 10.1002/1878-0261.70334 (PMC13558905; doi:10.1002/1878-0261.70334)
Supplement: Supplementary file 2 [file MOL2-9999-0-s002.pdf]

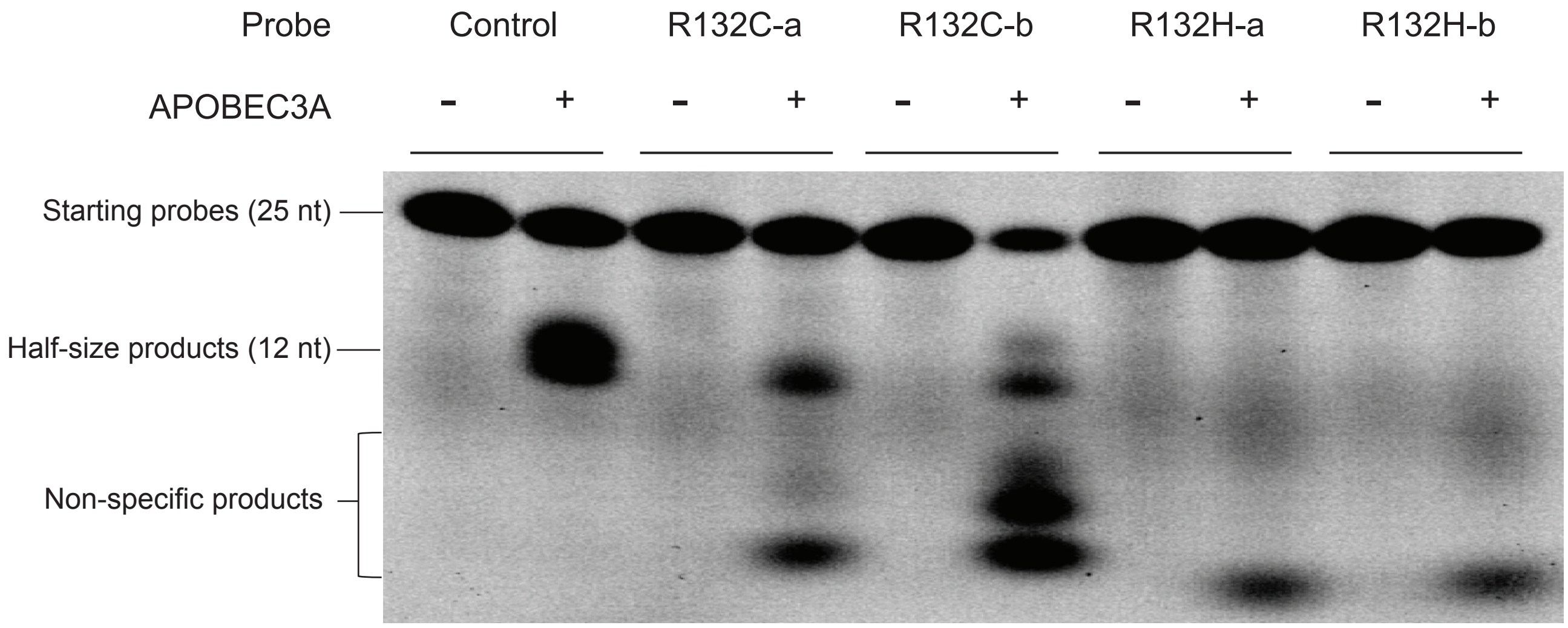
Probe
Control
R132C-a
R132C-b
R132H-a
R132H-b
APOBEC3A
- + - + - + - + - +
Starting probes (25 nt)
Half-size products (12 nt)
Non-specific products
